# Hyperglycemia and Lung Cancer—A Possible Relationship

**DOI:** 10.3390/diagnostics15060651

**Published:** 2025-03-07

**Authors:** Spasoje Popevic, Nikola Maric, Branislav Ilic, Slobodan Belic, Ivana Sekulovic Radovanovic, Sanja Dimic-Janjic, Mihailo Stjepanovic

**Affiliations:** 1 Faculty of Medicine, University of Belgrade, 11000 Belgrade, Serbia; ilicbranislav@yahoo.com (B.I.); belicslobodan@hotmail.com (S.B.); sanjadimicjanjic@gmail.com (S.D.-J.); mihailostjepanovic@gmail.com (M.S.); 2 Clinic of Pulmonology, University Clinical Center of Serbia, 11000 Belgrade, Serbia; nikolamaric1994@gmail.com (N.M.); ivanasekulovic@yahoo.com (I.S.R.)

**Keywords:** lung cancer, hyperglycemia, molecular pathways, treatment

## Abstract

Glucose is the main source of energy in human cells. Elevated levels of glucose are one of the most common metabolic disorders, and it has been shown to have a significant, mostly negative, effect on multiple chronic and acute diseases. Lung cancer remains one of the biggest challenges for treatment in modern medicine, with a high prevalence, incidence and mortality. Hyperglycemia is not uncommon in patients with lung cancer; however, it is usually overlooked. Patients with unregulated glycemia and lung cancer have been shown to have worse outcomes, reduced therapeutic effect and more complications during treatment. Studies have identified multiple molecular pathways common in both hyperglycemia and lung cancer; however, no clear correlation has been identified. By understanding these signaling pathways, we can influence the outcome therapeutically and thereby improve the survival of patients with lung cancer.

## 1. Introduction

Hyperglycemia and its role in cancer, particularly lung cancer, have become a subject of growing interest. The impact of metabolic dysregulation, along with complex metabolic and inflammatory pathways, on tumor behavior and treatment outcomes remains an area of active investigation.

In this narrative review, we summarize current knowledge from oncology, endocrinology and related fields to explore the interplay between hyperglycemia and lung cancer. By highlighting the latest research findings and relevant evidence, we examine its potential causal relationship with disease progression and therapeutic response.

The aim of this paper is to provide a well-rounded perspective and deeper understanding on how hyperglycemia may contribute to lung cancer pathophysiology and patient outcomes. This approach allows for a broader discussion of key findings while offering insights that may guide future research and clinical practice.

## 2. Materials and Methods

We conducted a comprehensive literature search using the PubMed database, with no restrictions on the initial publication dates of the included papers, covering studies from inception to January 2025. In this review, we evaluated peer-reviewed studies published in English, including prospective and retrospective studies, systematic reviews, and meta-analyses.

Our search strategy combined lung cancer-related terminology with terms related to hyperglycemia and metabolic dysregulation, specifically within title fields. Additionally, we identified additional relevant articles through the manual reference-checking of the included studies.

### 2.1. Understanding Glucose Metabolism in Health and Disease

Glucose is an organic compound that represents the main source of energy in the human body. Exogenic glucose is ingested as a mono-, di- or polysaccharide, and its degradation starts with saliva, as only monosaccharides can be absorbed in the small intestine. Glucose is freely transported in blood to virtually all cells that require energy. The excess glucose is stored as glycogen, a polymer of glucose, that can be degraded to glucose in states of fasting; organs rich in glycogen are the liver and muscles. With significant excess of glucose, when glycogen capacities are filled, glucose can be converted into fat via a process called liponeogenesis. On the contrary, in states of severe or prolonged fasting, when glycogen deposits are expended, glucose can be synthetized from other precursors such as glycerol, lactate, pyruvate or certain amino acids in a process called gluconeogenesis. Glucose can be metabolized by two main process pathways in order to provide energy: aerobic and anaerobic. Both processes are essentially a series of chemical reactions whose end goal is to create ATP, as it is the “main energy molecule” for all procedures in cell metabolism. Both pathways initially start as anaerobic, as glucose is degraded to pyruvate with minimal ATP production; if enough oxygen is present, pyruvate will enter the citric acid (Krebs) cycle, with a theoretical net yield of 32 ATP molecules from a single glucose molecule. On the other hand, if the metabolism is occurring in an anaerobic environment, pyruvate will be further reduced to lactate, with significantly lower production of ATP [1,2].

The main cellular receptors for glucose belong to two main groups: sodium-glucose linked transporters (SGLTs) and glucose transporters (GLUTs). SGLTs are sodium-dependent transporters, located primary on the intestinal epithelium and renal tubular cells. Their main role is to (re)absorb glucose into the blood. In the intestinal epithelium, ATP is utilized to essentially pump out sodium in order to create gradients to absorb glucose. On the other hand, due to already existing gradients of sodium in primary urine, the reabsorption of glucose is passive in renal tubules (as long as the glucose concentration in blood is less than 12 mmol/L). GLUT receptors have four subtypes and do not require sodium and ATP for function, as glucose is transported via passive transport. GLUT-1 located in the liver, pancreas and red blood cells allows for insulin/glucagon feedback mechanism, the main mechanism for the regulation of glucose level in blood. GLUT-2 is the main receptor for the uptake of glucose by the endocrine part of pancreas. GLUT-3 is located primarily in the central nervous system and has the highest affinity for glucose, preserving the brain in the states of fasting. GLUT-4, a high-glucose affinity receptor, is preset in adipose and muscle tissue, and is only active when stimulated by insulin, meaning it allows for storing glucose in states of elevated blood sugar (Table 1).

There are multiple hormones that influence the level of blood glucose, either by elevating or reducing its level. Insulin, via GLUT-4 receptors, and somatostatin, via the suppression of glucagon, gastrin and pituitary hormones, lead to reduction in glucose levels. Glucagon, gastrin, cortisol, epinephrine, thyroxin, growth hormone and adrenocorticotropin hormone all stimulate gluconeogenesis, increase fatty acids release from adipose tissue and antagonize insulin effect [1].

One of the most common pathological causes of hyperglycemia is diabetes mellitus (DM). Hyperglycemia in DM is caused either through the reduction in blood insulin levels (as mostly seen in the autoimmune destruction of β cells in the pancreas; T1DM) or through the reduction in the insulin effect on insulin target cells (T2DM). Patients typically present with hyperglycemia and an inability of peripheral tissues to utilize glucose, leading to the activation of alternative metabolic pathways that break down proteins and lipids for energy (Image 1). Prolonged hyperglycemia leads to the overall increase in oxidative stress in the human body and increases the rate of atherosclerosis, both leading to permanent organ damage (retinopathy, nephropathy, neuropathy, microangiopathy, coronary disease). Treatment options can be separated into two large groups: nonpharmacological and pharmacological. An increase in physical activity, a reduction in body mass, an appropriate diet, night sleep and the avoidance of stress are considered nonpharmacological measurements that should be implemented, regardless of the type (T1DM or T2DM). As per pharmacological treatment, insulin for T1DM and metformin for T2DM, are still first-line treatments [3]. In T2DM, an important clinical feature when determining treatment is the presence of cardiovascular diseases/complications, which, if present, require further treatment, either with sodium-glucose cotransporter-2 (SGLT2) inhibitors or glucagon-like peptide 1 (GLP-1) agonists [3].

### 2.2. Lung Cancer

**Figure 1:**
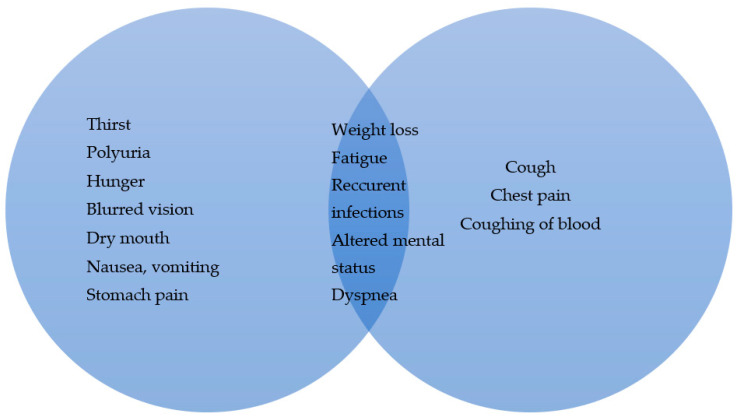
Signs and symptoms of hyperglycemia and LC, with overlap.

Lung cancer (LC) is one of the most prevalent and lethal types of cancer worldwide. The usual clinical presentation is unspecific and consists of coughing, fatigue, chest pain and weight loss; if metastasis is present, initial presentation can be specific to the afflicted organ (Figure 1). For full diagnostics, besides pathohistological verification, a radiographic evaluation is required in order to precisely determine the tumor, nodule, metastasis stage (TNM). Therapeutic modalities depend on the type of LC and its stage and can consist of operation, radiotherapy, chemotherapy, immunotherapy, molecular therapy or a combination of multiple agents, either concurringly or in sequence.

## 3. Molecular Pathways Common in Both DM and LC

### 3.1. Interleukin 6 (IL-6)

IL-6 is a proinflammatory cytokine produced by macrophages in the earliest stages of local inflammation. When secreted, the primary target organ is the liver, where it causes the production of other proinflammatory agents—C reactive protein (CRP), fibrinogen, haptoglobin, serum amyloid A and α1antichymotripsin—and suppresses fibronectin, albumin and transferrin. IL-6 also causes the maturation and differentiation of cytotoxic CD8 T cells and Th17 CD4 T cells. On B cells, IL-6 stimulates the production of antibodies. If the cause of inflammation is unresolved, IL-6 stimulates fibroblasts, which further cause angiogenesis and fibrogenesis [4].

IL-6 regulates glucose homeostasis by stimulating bowel L cells as well as pancreatic α cells to produce and secrete glucagon-like peptide-1 (GLP-1), further increasing insulin secretion. Despite this effect, theories have been proposed stipulating that IL-6 leads to peripheral insulin resistance [5,6]; the role of IL-6 in T1DM has been more researched compared to the effect on T2DM. However, multiple studies have shown that the suppression of IL-6 leads to the improvement of insulin sensitivity and glucose metabolism, regardless of the type of DM [7,8,9].

IL-6 in LC has been found to be secreted by tumor stroma and has been shown to be correlated with a worse prognosis. It has been found that IL-6 stimulates the expression of transforming growth factor-β1-induced epithelial-to-mesenchymal transition (EMT) changes in cancer cells and reduces ABCG2 (the gene responsible for chemotherapy resistance) and anti-apoptotic protein expression. This has a dual effect: the increase in EMT increases the risk of metastasis, and the inhibition of anti-apoptotic proteins reduces the efficacy of chemotherapy [10,11,12]. A special place for IL-6 in LC treatment, specifically its inhibition, is in K-ras-positive non-small-cell lung cancer (NSCLC), as patients with a positive K-ras LC have significant inflammation in tumor stroma [13]. The suppression of IL-6 in these patients has shown not only direct inhibition for tumor neogenesis but also changes in the stromal profile into an antitumor one.

### 3.2. Transforming Growth Factor (TGF)–β

TGF-β is a family of cytokines expressed on virtually all cells in human organisms. Virtually all functions of the cell, such as metabolism, proliferation, differentiation, migration, migration and apoptosis, are regulated by TGF-β. In T1DM, TGF-β has a protective effect as it increases the tolerance of T cells regarding pancreatic cells. Additionally, hyperglycemia present in DM can cause EMT, via TGF-β1. TGF-β1 activates fibroblasts in the afflicted tissue and causes the buildup of the extracellular matrix [14,15]. The described fibrosis is key in the development of DM complications such as microangiopathy, nephropathy and retinopathy; however, the same damage is present in virtually all organs, including the lungs [14,16].

In LC, TGF-β has been shown to correlate with poor prognosis and early metastasis. An extracellular matrix rich in TGF-β can provide a good substrate for tumorigenesis, as TGF-β stimulates EMT, further reducing the ability of adhesion of cells, thus enabling easier migration. TGF-β also stimulates the differentiation of LC cells, via autocrine and paracrine effects, causing a positive feedback loop [17]. On normal epithelial cells, TGF-β has an inhibitory effect on their proliferation; the same effect is not only absent in LC cells but also has an opposite effect [18].

### 3.3. Hypoxia and Hypoxia-Inducible Factors (HIFs)

Hypoxia in any tissue causes an increase in anaerobic metabolism and consequentially reduces the local pH value, which increases oxidative stress. Depending on the tissue and the length of the hypoxia, the afflicted cells can go into apoptosis or necrosis. If hypoxia is not resolved, defensive mechanisms will be activated, such as the alteration of the gene expression, anaerobic glycolysis, neoangiogenesis and/or fibrosis. The main moderators for these mechanisms are called hypoxia-inducible factors (HIFs). HIFs are a group of signaling molecules whose primary objective is to maintain cellular oxidative homeostasis. Biochemically, an HIF consists of two subunits, an α subunit that is sensitive to hypoxia and a 1β subunit that is constitutively expressed. The α subunit has three isoforms: 1α (HIF-1) is present in all tissues, while 2α (HIF-2) and 3α (HIF-3) are tissue-specific, usually in hypoxia-sensitive tissues. In defensive responses, HIF-1 is the first and quickest to activate, followed by HIF-2, while the role of HIF-3 still requires further studies. The activity of HIF-1 is oxygen-dependent: at normal oxygen levels, HIF-1 is degraded by HIF prolyl hydroxylation, while in hypoxia, this enzyme is inhibited, which leads to the accumulation of HIF-1 and allows for its function. HIF-2 is active in slightly hypoxic environments, but also in environments with normal levels of oxygen, compared to HIF-1 [19].

Hypoxia is commonly found in DM. Accelerated atherosclerosis on small blood vessels damages the capillary exchange of gases and nutritive matter, and the reduced uptake of glucose leads to alternative metabolic pathways in the afflicted cells. When observing animal models, HIF-1 exhibits reduced activation in hypoxic tissues in individuals with DM. The main theory is that high glucose levels inhibit protein stabilization and function in hypoxic environments. Methylglyoxal, a product of anaerobic glycolysis, derived from pyruvic acid, has been shown to cause endothelial disfunction, and has been associated with increased oxidative stress, further stimulating anaerobic metabolism [20,21].

Due to a high metabolic rate and mitotic index, tumor stroma induces neoangiogenesis. Hoverer, the new blood vessels are immature and cannot compensate adequately for ischemia; the ischemia is further increased due to the in situ thrombosis of new blood vessels. Both HIF-1 and HIF-2 have been shown to have a significant place in LC pathophysiology [22,23]. HIF-1 has been found to be expressed significantly more in LC cells compared to healthy lung cells; in addition, squamous cell LC has been shown to have a higher HIF-1 expression compared to adenocarcinoma. HIF-1 binds to, and inhibits, survivin, a protein responsible for cell apoptosis; the inhibition of survivin leads to reduction in the apoptosis of LC cells. A study showed that patients with LC and a high HIF-1 expression are more likely to have lymph node affliction compared to those with low HIF-1 expression [23]. Further analysis has shown that HIF-1 induces the production of vascular endothelial growth factor (VEGF), allowing for the neoangiogenesis of immature blood vessels, which are more easily invaded by LC cells. Extracellular matrix metalloproteinase inducer CD147 degrades both intercellular connections and basal membrane, allowing for easier metastasis of LC cells; a positive correlation between HIF-1 and CD147 in LC cells has been shown. High HIF-1 expression has also been shown to be correlated with the size of the primary tumor and with the higher stages of LC [24].

### 3.4. Platelet-Derived Growth Factor (PDGF)

Platelet-derived growth factors (PDGFs) are a group of proteins that have a crucial role in angiogenesis. They are synthesized by platelets, smooth muscle cells, activated macrophages and endothelial cells, and the factors that activate PDGF are hypoxia, hyperglycemia, thrombin and other cytokines. In addition, autocrine stimulation with PDGF is noted in tumor cells. A special role of PDGF is present in lung growth, as it causes alveolar septal formation, alveogenesis and alveolar myofibroblast development. The activation of PDGF occurs when four PDGF polypeptides (A, B, C and D) are bound to form five functional growth factors (PDGF-AA, -BB, -AB, -CC and –DD). Functional growth factors are then bounded to adequate receptors (PDGFR-α and PDGFR-β), which activates downstream signaling inside of the cell [25,26,27].

The main correlation between DM and PDGF is hyperglycemia and oxidative stress. In patients with DM and obesity, genetic testing has shown an elevated expression of PDGFA gene. In patients with any form of hyperglycemia, the downregulation of PDGF-BB and PDGF-C has shown a significant impairment of vascular remodeling and consequent reduction in blood recirculation [28].

In LC with poor prognosis, a higher expression of both PDGFRα/β and PDGF-A/B has been found. In contrast, high PDGFR-α expression in stromal cells was an indicator of increased survival, while high PDGFR-β expression was associated with poor survival. The amplifications of PDGFR-A and PDGF-C genes have been found in NSCLC, leading to a form of independency, as these tumor cells have a higher number of receptors on its surface, leading to a relatively higher sensitivity for PDGF stimulation. Furthermore, the paracrine expression of PDGF-α and -C has been shown to increase the activity of fibroblasts, which then in turn stimulate the production of tumor stroma [29,30,31].

Disfunction in the previously mentioned pathways (IL-6, TGF-β, HIF, PDGF) in both hyperglycemic states and LC is important; however, there have been no clinical trials, up to the time of the writing of this article, that have attempted to define correlation and casualization. One of the possible reasons lies in the fact that it is difficult to isolate only one pathway, given that, as mentioned earlier, there are common mechanisms that induce several pathways. Another reason is the large number of causes of hyperglycemia, and it is difficult to establish a standardization for further investigation.

## 4. Effects of Hyperglycemia on Lung Cancer Treatment

Seeing that there are multiple common pathways between hyperglycemia in DM and LC, one could presume that there could be a certain specificity in both treatment and complications. Different studies have analyzed preexisting DM as one of prognostics factors in patients with LC. In one retrospective study, preexisting DM was shown to be an independent and significant prognostic factor for worse survivability in locally advanced NSCLC [32]. Another study, however, showed that preexisting DM caused worse survivability only in female LC patients [33]. A proper glycemic control (hemoglobin A1c (HbA1c) <7%) has been shown to have a positive outcome in patients with NSCLC patients who underwent surgery treatment; it is proposed that well-controlled DM reduces risk for uncontrolled inflammation in LC itself, and allows for proper recovery from surgery [34]. Also, the association between hyperglycemia regarding the level of HbA1c and locoregional recurrence-free survival in patients with limited-stage small-cell lung cancer treated with radical radiotherapy (RT) was examined based on the cut-off value for HbA1c of 6%, patients were divided into two groups, a low- and high-HbA1c group. Multivariate analysis showed that the level of HbA1c is a significant prognostic factor for locoregional recurrence-free survival and that in the low-HbA1c group, 1-, 2- and 3-year locoregional recurrence-free survival rates and distant metastasis-free survival rates are significantly higher compared with the high-HbA1c group (*p* < 0.01) [34]. Besides surgery, the significance of DM in patients treated with radiotherapy is undeniable. A study that compared the development of post-radiation pneumonitis between patients with and without DM showed that patients with DM have a higher risk for developing post-radiation pneumonitis [35]. It should be noted that there was no difference between irradiation area, tumor pathological type, degree of differentiation and classification of malignant tumor (TNM) stage; there was, however, a significant difference between body weight, age and hypertension. Another study analyzed DM, its laboratory finding and the development of post-radiation pneumonitis in patients with LC. The presence of DM, elevated glycemia and HbA1c are significant factors for the development of higher grades (≥3) of pneumonitis and require intensive treatment of DM [36].

## 5. Hyperglycemia and Chemotherapy

Patients with LC undergoing chemotherapy must monitor their blood glucose levels and blood glucose fluctuation, as hyperglycemia alone can affect the curative effect of chemotherapy.

NSCLC patients with large variations in glucose levels and, in general, high blood glucose levels have poor prognosis and increased mortality, indicating that DM and hyperglycemia effectively controlled may present an opportunity for prognosis improvement and controlling disease progression [37,38].

Hyperglycemia alters the response to chemotherapeutic drugs, can increase toxicity and incidence of associated complications and can contribute to reduced chemotherapeutic treatment efficacy by affecting the cellular innate immunity. It may promote the development of a more malignant phenotype of cancer cells and lead to drug resistance. Followed by high serum insulin levels, hyperglycemia can induce the proliferation of cancer cells (glucose-hungry cells) through different mechanisms, consequently supporting tumor cell growth [38]. One study confirmed higher incidence of adverse reactions (including phlebitis, gastrointestinal reactions, oral ulcers and liver and kidney damage) in patients with larger blood glucose fluctuations who were treated with chemotherapy [39].

It is important to emphasize that patients undergoing chemotherapy are at an increased risk of developing new blood glucose regulation issues due to the impact of chemotherapy on metabolism and glucose levels, which can lead to reduced clinical efficacy and worse overall prognosis [40]. Glucose metabolism disorders may occur after administrated chemotherapy, leading to a significant increase in blood glucose levels. Some chemotherapeutic agents damage insulin β cell, impact insulin synthesis and secretion, block blood glucose control and induce secondary DM. Long-term hyperglycemia can suspend chemotherapy and affect the quality of life of patients [40].

Corticosteroids are frequently used in the treatment of LC patients, especially when undergoing highly emetogenic chemotherapy. Corticosteroids cause alterations in glucose metabolism through insulin resistance and reduced insulin secretion. If used frequently, in high doses and intravenously, they can cause hyperglycemia and prolonged poor blood glucose regulation [41]. When associated with other coexisting causative agents of hyperglycemia, corticosteroid treatment can exacerbate already compromised glucose regulation and may induce hyperglycemia that is challenging to manage, potentially leading to reductions in or the interruption or cessation of chemotherapy doses.

It is recommended that blood glucose levels should be monitored carefully, especially during inductive chemotherapy in patients at high risk of hyperglycemia. While managing high blood glucose levels and adjusting the insulin dosage, it is advised to simultaneously work on preventing the side effects of chemotherapy, such as chemotherapy-induced nausea and vomiting, severe dehydration and water and electrolyte disorders, in order to prevent further metabolism disorders [40].

## 6. Tobacco-Induced Hyperglycemia and LC

Tobacco smoking is an important risk factor for developing LC, and exposure to tobacco is by far, no matter the differences in intensity in smoking or preferred type of cigarettes, the most linked to contributing approximately 90% of all lung cancers [42,43]. Nevertheless, a significant number of patients continue to use tobacco despite being diagnosed with LC and given recommendations, even during ongoing treatment.

Tobacco carcinogens can induce and promote tumor activities in LC cells [44]. Exposure to these carcinogens can also contribute to developing metabolic syndrome and various medical conditions, such as elevated levels of blood glucose and serum triglyceride. Hyperglycemia has been involved in the etiology of tumor formation and progression affecting tumor-associated macrophages, one of the main types of stromal cells in the tumor microenvironment, related to cancer progression [45]. There are several studies that have investigated the association between hyperglycemia as part of the metabolic syndrome and the risk of various types of cancer, given that the metabolic syndrome is a serious public health problem with increasing prevalence. One of the largest studies that examined the relationship between metabolic syndrome and the risk of developing LC is a study conducted on the UK population, with over 330,000 patients and a median follow-up of about 11 years. In the study population, a higher incidence of LC was observed in the group of patients with metabolic syndrome, and risk was associated with all components of metabolic syndrome; specifically, in patients with hyperglycemia, the hazard ratio (HR) was 1.3 (95% CI, 1.16–1.45). The HR was higher in women than in men (1.4 vs. 1.23), but without statistical significance. The association between HbA1c levels and LC was also shown, but it had a U-shape, with the highest risk for both high and low HbA1c levels and the lowest risk around 32 mmol/mol (5.1%). This connection can be explained by the impact of hyperglycemia, high insulin levels and insulin resistance on the activation of the IGFR1-IR-PI3K-AKT-mTOR pathway, which is considered a cause of carcinogenesis [46].

A recent study examined the impact of chronic exposure to tobacco carcinogens on developing metabolic syndrome, lung cancer and the correlation of tobacco-mediated metabolic syndrome in lung cancer progression. The study was conducted on mice, exposing them to primary tobacco smoke components, 4-(methylnitrosamino) −1-(3-pyridyl) −1-butanone and benzo[a]pyrene (nicotine-derived nitrosamine ketone (NNK) and BaP; NB). The results of this study showed that exposure to tobacco smoke promotes the progression of lung cancer through a key mechanism involving cancer–stroma communication facilitated by NB-mediated systemic and local complications in glucose metabolism. Their study findings showed that NB exposure enhances glucose consumption in monocytes/macrophages via a two-step pathway, the BaP/aryl hydrocarbon receptor-mediated transcriptional upregulation of GLUT 1 and GLUT3 and NNK/nicotinic acetylcholine receptor-triggered membrane translocation of the transporters, leading to the upregulation of genes involved in tumor infiltration and protumor activities. Not excluding other potential mechanisms, they formulated the hypothesis that NB exposure promotes lung cancer progression by inducing hyperglycemia in tumor macroenvironments, which preprograms monocytes/macrophages in tumor microenvironments to acquire protumoral phenotypes. Data collected from this study indicated that paracrine IGF2/IR/NPM1/PD-L1 signaling, facilitated by the NB-induced dysregulation of glucose levels and metabolic reprogramming of macrophages, contributes to tobacco smoking-mediated LC progression [47,48].

## 7. Hyperglycemia, Risk of Infection and Poor-Outcome Treatment of LC Patients

LC patients are at greater risk of developing infections, due to altered immune response, especially in patients treated for chronic obstructive pulmonary disease as well, where chronic inflammation and associated lung pathology are present.

When infection occurs in LC patients, it is necessary to provide adequate treatment, given the potential complications of infections. It is always recommended to provide early detection of various pathogens, especially in LC patients, as they are susceptible to attacks from both opportunistic and various aggressive pathogenic bacteria and viruses. These patients are highly dependent on a number of treatments, including immunotherapy, which can lead to the modulation of the systemic immune response. Infectious agents may facilitate the development of an inflammatory environment prone to LC initiation and progression, as well as the response to therapy [49].

One of the factors that can contribute to developing complications related to ongoing infection is hyperglycemia, which can be caused by various factors. Whether it is caused by the tumor itself, oncologic or symptomatic therapy or accompanying comorbidities, hyperglycemia can alter infection treatment outcomes, prolong treatment and delay specific ongoing oncologic therapies.

Since LC can be considered to develop immunocompromised status in patients, it may promote tuberculosis infection or result in the reactivation of latent tuberculosis. Tuberculosis (TB) can also occur from granulomas microenvironment deregulation due to regional tumor peptides, antigens, cytotoxic chemotherapy or radiotherapy, allowing mycobacteria to proliferate [50,51].

In considering that TB and LC can coexist, especially in endemic areas, the treatment of tuberculosis during systemic anticancer therapy can be challenging [52,53].

Referring to treatment of TB, one prospective study showed that patients with transient hyperglycemia or DM were most likely to experience treatment failure [53]. Another study reported that hyperglycemic patients with TB were more likely to have various types of pulmonary lesions such as cavities, alveolar infiltrates, and fibrous tracts compared to euglycemic patients, given that the rate of pulmonary lesions is related to the level of glycemic control—the poorer the glycemic control, the higher the rates of pulmonary lesions [54]. With previously impaired lung architectonics due to the presence of LC, additional lung lesions that are challenging to treat can play a significant role in worsening respiratory symptoms and can result in the deterioration of the general health status of patients, causing an overall poor life expectancy.

LC patients with known metastasis in the pleura, leading to chronic malignant pleural effusion, are presumed to be at greater risk of developing severe infections. Patients with LC and known hyperglycemia or DM are at potential risk for respiratory infections progressing to pleuropneumonia and pleural empyema, often requiring frequent thoracentesis or other invasive procedures. A retrospective study showed that up to 30.1% of all purulent thoracic surgical diseases occur in the context of unregulated glucose levels or DM [55]. Managing pleural empyema and lung abscesses can be particularly challenging, as providing the appropriate treatment without impairing the patient’s quality of life is difficult, especially when it results in delays to oncologic therapy.

With the improvement of LC treatment, analyzing the patient’s mental state is increasingly in focus. Anxiety, depression, sleep and sexual disorders are frequently found in patients with any form of cancer [56]; however, in patients with lung cancer, these states can have an impact on overall outcome [57]. On the other hand, metabolic syndrome has been associated with increased risk for stress-based disorders, such as depression and anxiety [58,59]. In patients with good performance status, proper glycemic control, especially with physical activity, can lead to a significant reduction in psychological symptoms [60,61].

## 8. Conclusions

Both hyperglycemia and LC are medical problems that are on the rise. Despite many attempts to fully understand any and all possible correlations and causal relationships between these diseases, much still remains unknown. However, some things have been proven; unregulated glycemia is associated with a poor outcome in LC treatment. Further trials are needed in order to discover any potential pathways that will help us better understand the relationship between LC and hyperglycemia.

## Figures and Tables

**Table 1:** Main receptors for glucose in human body.

Transporter	Most Common Location	Type of Transport	Function
SGLT	Renal tubules, intestinal mucosa	Secondary active transport	(Re)absorption of glucose
GLUT 1	Ubiquitous	Facilitated diffusion	Basal glucose uptake
GLUT 2	Pancreatic β cells, hepatocytes, intestinal mucosa, kidney	Facilitated diffusion	Regulation of blood glucose levels
GLUT 3	Nervous system	Facilitated diffusion	Maintains glucose uptake in nervous system, regardless of blood glucose levels
GLUT 4	Skeletal and cardiac muscle tissue, adipose tissue	Facilitated diffusion	Insulin-dependent, regulates glucose uptake in hyperglycemia

## Data Availability

Data is contained within the article.
